# Seasonal variability in global industrial fishing effort

**DOI:** 10.1371/journal.pone.0216819

**Published:** 2019-05-17

**Authors:** Jérôme Guiet, Eric Galbraith, David Kroodsma, Boris Worm

**Affiliations:** 1 Institut de Ciència i Tecnologia Ambientals (ICTA-UAB), Universitat Autònoma de Barcelona, Barcelona, Spain; 2 Department of Atmospheric and Oceanic Sciences, University of California Los Angeles, Los Angeles, California, United States of America; 3 Institució Catalana de Recerca i Estudis Avançats (ICREA), Pg. Lluís Companys, Barcelona, Spain; 4 Global Fishing Watch, Washington DC, United States of America; 5 Biology Department, Dalhousie University, Halifax, Nova Scotia, Canada; Bangor University, UNITED KINGDOM

## Abstract

Human beings are the dominant top predator in the marine ecosystem. Throughout most of the global ocean this predation is carried out by industrial fishing vessels, that can now be observed in unprecedented detail via satellite monitoring of Automatic Identification System (AIS) messages. The spatial and temporal distribution of this fishing effort emerges from the coupled interaction of ecological and socio-economic drivers and can therefore yield insights on the dynamics of both the ecosystem and fishers. Here we analyze temporal variability of industrial fishing effort from 2015-2017 as recorded by global AIS coverage, and differentiated by fishing gear type. The strongest seasonal signal is a reduction of total deployed effort during the annual fishing moratorium on the numerically-dominant Chinese fleet, which occurs during boreal summer. An additional societally-controlled reduction of effort occurs during boreal winter holidays. After accounting for these societal controls, the total deployed effort is relatively invariant throughout the year for all gear types except squid jiggers and coastal purse seiners. Despite constant deployment levels, strong seasonal variability occurs in the spatial pattern of fishing effort for gears targeting motile pelagic species, including purse seiners, squid jiggers and longliners. Trawlers and fixed gears target bottom-associated coastal prey and show very little overall seasonality, although they exhibit more seasonal variation at locations that are further from port. Our results suggest that societal controls dominate the total deployment of fishing effort, while the behavior of pelagic fish, including seasonal migration and aggregation, is likely the most prominent driver of the spatial seasonal variations in global fishing effort.

## Introduction

The seasonal spatio-temporal variability of prey plays a large role in the behavior of marine top predators, influencing their foraging strategies [1] and driving them to undertake large migrations [2, 3]. Over the last century, humans emerged as a globally-dominant predator of wild marine fish, due primarily to large advances in fishing technology [4]. The high efficiency of modern industrial fishing vessels allows humans to capture a high fraction of their targeted fish, exerting a median mortality rate on adult fish that is one order of magnitude larger than any other natural top predator [5]. The industrial fishery exploits many thousands of local fish stocks, each of which can vary according to its own seasonal rhythms, while the millions of people employed in fishing and fish processing are also subject to societal and economic dynamics that could independently influence the emergent behavior of the fishery. In view of the vast scope and advanced technological state of the global fishing enterprise, we asked to what extent predation by humans follows natural seasonal variations, as opposed to the socio-economic influences that drive many other industries.

The seasonal interactions between the environment and fishers is often locally well known at the level of stocks [6–8] or for ecosystems [9, 10], for both industrial [11, 12] and small-scale fisheries [13, 14]. Models have been developed in order to analyse how natural and socio-economic drivers shape the seasonal dynamics of fishing effort, for single- [15–17] and multi-species fisheries [18]. In addition, tools that forecast the seasonal dynamic of marine resources are now being implemented to assist management [19]. However, a global perspective on fishing seasonality has remained absent, because spatially explicit maps of global fishing effort over different seasons and years have been unavailable until recently.

Reconstructions of the global fishing effort distribution using gravity models have been available for some time [20–22], but although very useful for the study of interannual effort distributions, these model-based reconstructions lack sub-annual temporal resolution. Recent improvements in satellite coverage and artificial intelligence analysis have now made it possible to follow the fishing activity of industrial vessels through the monitoring of their Automatic Identification Systems (AIS), a security device implemented to prevent collisions. The Global Fishing Watch (GFW) project [23] has collected these data since 2012, and developed convolutional neural network algorithms to distinguish fishing from non-fishing activities and identify the effort distribution for different fishing gear types at high spatial and temporal resolution [24, 25]. AIS has inherent limitations that restrict coverage largely to industrial vessels fishing offshore, and thus is less inclusive than some model-based reconstructions [20, 21]. Yet it complements earlier work by providing a direct empirical view of the seasonal variation of fishing effort worldwide.

The main objective of this study is to describe the seasonality of fishing effort by gear type in the GFW dataset, including variation in the total effort, spatial characteristics, and variation as a function of the distance to nearest port. We subsequently discuss the first-order mechanisms that would be expected to contribute to ecological versus socio-economic drivers of the observed seasonality.

## Materials and methods

### Data sources

Until very recently, most publicly-available estimates of global fishing effort relied on data aggregated at the country level [22, 26], from which spatial distributions were estimated using assumptions on the attractiveness of fishing grounds [20, 21]. Global Fishing Watch (GFW) has recently revolutionized the availability of global effort data by analyzing the positions of boats tracked since 2012 using the Automatic Identification Systems (AIS). AIS are safety devices which are used to avoid collisions between ships at sea. They broadcast information about their position and direction every 2 s to 3 min. This information can be received by ships in the neighborhood and by receivers in line of sight but also by low-orbit satellites. While regulations for fishing vessels vary by flag state, AIS is a compulsory device on almost all fishing vessels larger than 300 gross tons, and it is becoming mandatory or otherwise widely adopted for many smaller vessels as well. GFW collects these data to map the distribution of AIS-tracked fishing vessels, at a high temporal resolution, providing a transparent image of global fishing activities [23, 27].

The AIS messages provide a series of positions for each vessel, which are then processed by two convolutional neural networks to characterize the vessel and detect periods of fishing activity [25]. The first model characterizes fishing vessels and their gear type with 95% accuracy when compared to labelled data. The second model identifies the periods of fishing activity and provides effort distributions for each gear type, namely trawled gears, fixed gears, drifting longlines and purse seines with respectively 96%, 97, 94 and 95% accuracy [25]. For squid jigger vessels the fishing activity is detected when these are standing still for more than 4 hours at night, more than 10 nm from shore. A sixth category, ‘other’, includes pole and line, trolled, and other less common types of gear. We will mainly focus on the first five categories of gears. When the fishing vessel sizes are taken into account, the GFW effort accounts for an estimated 50-70% of the total energy used by all fishing vessels beyond 100 nm from land [25].

The GFW estimate of effort used here is expressed as the number of fishing-vessel-days per 1.0° grid cell. The number of fishing-vessel-days in each grid cell is determined at daily resolution by summing the number of vessels fishing at least once during a given day. The aggregation of fishing effort to a daily resolution is intended to smooth out differences in the proportion of time spent looking for prey vs. actively fishing among different gear types. For example, a purse seiner targets schools of fish and will typically spend a large fraction of time searching before deploying a net, whereas longliners deploy their gear for several hours each day with little or no search time. We chose a resolution of 1.0° (*i.e*. dimensions of roughly 110x110 km at the equator) because it corresponds to the approximate spatial dimension of a longline set (the most commonly used geartype in the open ocean) and the daily scale of movement of many industrial fishing vessels. Using a higher resolution would impair the detection of interannual seasonal signals due to low absolute vessel presence in each grid cell, while lower resolutions impair the identification of fine seasonal spatial patterns. The spatial resolution of 1.0° is of the same order of magnitude as previous model-based methods [20, 21], but temporal resolution is by day instead of year, and is directly observed rather than being reconstructed.

Although the GFW coverage is relatively complete for the high seas at the global scale, there is significant variability in the extent of coverage in coastal waters and at regional scales. In general, AIS data lacks small scale fisheries and is biased toward industrial fisheries and wealthier nations. The vast majority of large vessels (>24m), which are more likely to carry AIS, are from upper middle income or high-income countries [28]. AIS regulations exacerbate this bias, as they are generally stricter in wealthier nations, with some of the strongest regulations being in European Union, where all fishing vessels 15 m or larger are mandated to broadcast AIS. In contrast, only a small fraction of the fleets of many developing nations are broadcasting, such as those in Africa, south Asia, Southeast Asia, or Latin America. An important region particularly lacking AIS data is Southeast Asia, including Indonesia, where AIS use by fishing vessels is low and where reception from satellites is poor due to signal interference from abundant non-fishing vessels using AIS in the region [25]. In addition to coverage issues, AIS signals can be manually turned off, impairing the estimation of global effort for some fleets. Because of these limitations the dataset may be less inclusive than other effort estimates [20, 21]. Despite these caveats, the global scope of the GFW dataset and high temporal resolution makes it an unprecedented source of information with which to assess the main drivers of fishing seasonality at a global scale. We consider GFW effort distributions from 2015 through 2017, selecting only vessels that were continually active throughout the 3 years to remove any trend due to the gradual increase in adoption of AIS device.

### Effort distribution

In order to analyze seasonal patterns of fishing effort distribution we consider *E*_*g*_, the monthly globally-integrated effort by gear type *g*, divided by the number of days of the month (in fishing days per day). The total global mean daily effort in any month is given by *E* = ∑_*g*_ *E*_*g*_ = *E*_*Trawled*_ + *E*_*Fixed*_ + *E*_*Drifting*_ + *E*_*Purse*_ + *E*_*Jigger*_ + *E*_*Other*_, which includes trawled gears *E*_*Trawled*_, fixed gears *E*_*Fixed*_, drifting longlines *E*_*Drifting*_, purse seines *E*_*Purse*_, squid jigger gears *E*_*Jigger*_ and other gears *E*_*Other*_. The effort of purse seiners *E*_*Purse*_ is split between coastal, *E*_*Purse* (*coastal*)_ and high seas purse seiners, *E*_*Purse* (*highseas*)_, where we define high seas purse seines as purse seine vessels larger than 40 m whose average fishing activity is more than 50 nm from shore.

We also describe $e_{g}^{\left(i,j\right)}$, the monthly local effort. Like *E*_*g*_, the $e_{g}^{\left(i,j\right)}$ is defined as the total effort per month for gear *g*, divided by the number of days of the month, but is for a single grid cell at longitude *i* and latitude *j*. We analyze the data at three different resolutions of longitude and latitude (*i*, *j*): 0.5°, 1.0° and 4.0°. This repetition tests the robustness of the results, since the presence/absence of effort in a grid cell depends on the size of the grid cell. We calculate the spatial distribution of yearly averaged mean local monthly efforts ${\bar{e}}^{\left(i,j\right)}=\sum_{g}{\bar{e}}_{g}^{\left(i,j\right)}$, from 2015 to 2017, to quantify the distribution of fishing activity.

### Effort seasonality

Globally, the seasonality of integrated fishing effort *E* for a given month is revealed by the relative variation of effort $E/\bar{E}$, where $\bar{E}$ is the mean effort from 2015 through 2017. The seasonality of the global effort per gear type *g* is determined the same way, $E_{g}/{\bar{E}}_{g}$. The standard deviation $sd(E_{g}/{\bar{E}}_{g})$ is an indicator of the intensity of the seasonal cycle.

Locally, the seasonality of fishing effort is described with different indicators. A simple metric for the intensity of the local seasonal cycles is provided by the standard deviation $sd(e^{\left(i,j\right)}/{\bar{e}}^{\left(i,j\right)})$, where ${\bar{e}}^{\left(i,j\right)}$ is the inter-annual mean monthly effort from 2015 to 2017. We also adopt a seasonality indicator for each (*i*, *j*) location, to quantify the evenness of the seasonal effort distribution [29]:

$$SI^{\left(i,j\right)}=\frac{1}{{\bar{e}}_{a}^{\left(i,j\right)}}\sum_{n=1}^{12}\left|e_{n}^{\left(i,j\right)}-\frac{{\bar{e}}_{a}^{\left(i,j\right)}}{12}\right|,$$ (1)

where ${\bar{e}}_{a}^{\left(i,j\right)}$ is the mean annual effort and $e_{n}^{\left(i,j\right)}$ the monthly effort. The values of *SI*^(*i*, *j*)^ vary between 0 and 1.83, where values lower than 0.4 reflect even distributions of fishing effort over the year, and values higher than 1.0 reflect compression of effort within a short interval of the year.

The distance from fishing grounds to port may also vary seasonally. Although we cannot link each observed fishing event to the vessel’s corresponding home port, we provide a global perspective on variations in the remoteness of fishing grounds by computing the mean local relative standard deviation of monthly effort per distance bins *b*, $\sum_{(i,j)\in b}\left(sd\left(e_{g}^{i,j}/{\bar{e}}_{g}^{i,j}\right){\bar{e}}_{g}^{i,j}\right)/\sum_{(i,j)\in b}\left({\bar{e}}_{g}^{i,j}\right)$. In order to remove noise we discard distance bins for which the summed annual effort corresponds to less than 0.1% of the total annual effort. For each ocean cell (*i*, *j*), the distance to port is interpolated at its center from a 1/100° map developed by GFW (see S1 Fig) [23].

## Results

### Global effort seasonality

**Fig 1:**
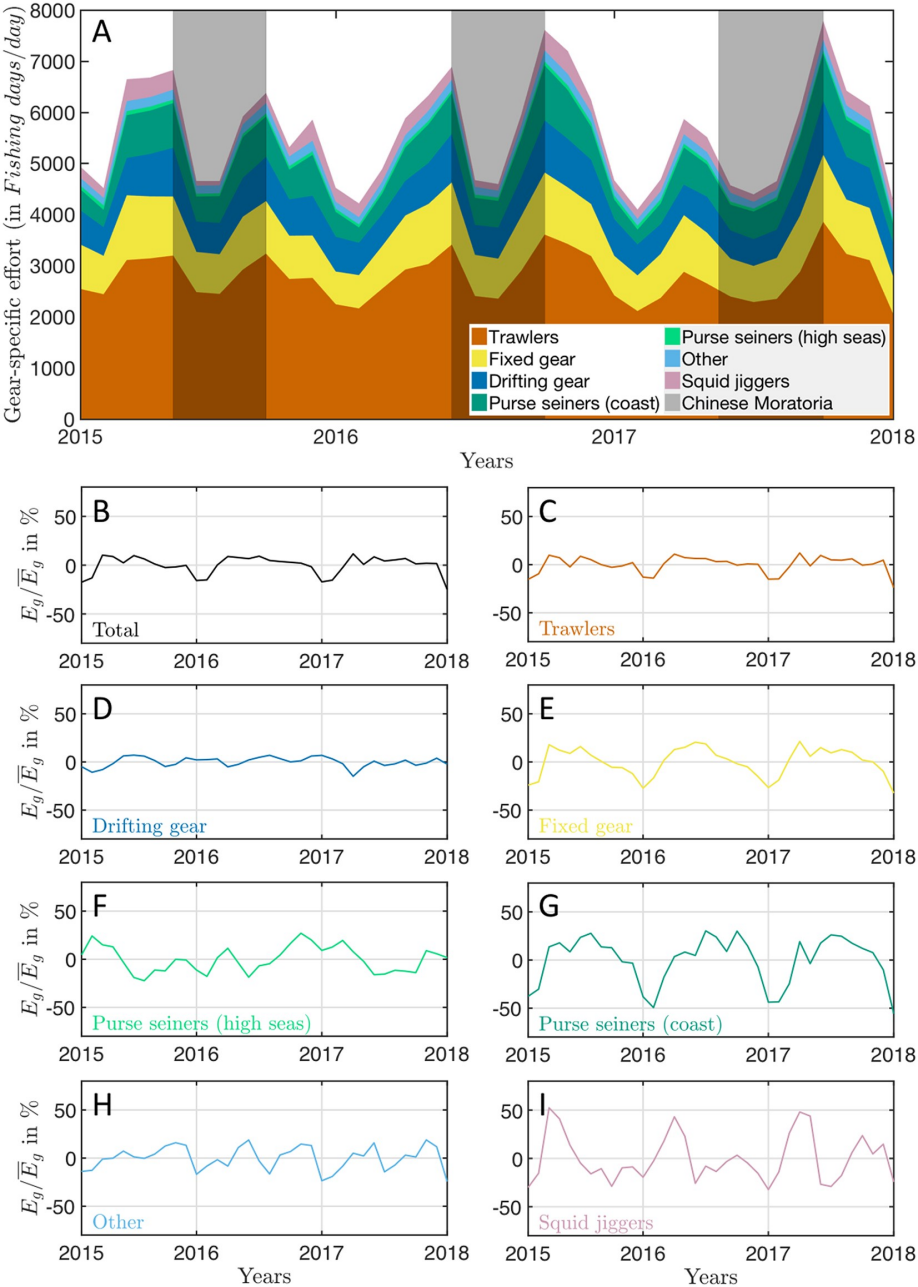
Summed global mean monthly fishing effort per gear type. (A) monthly mean number of fishing days normalized by the number of days for each month *E*_*g*_, reflecting the mean number of fishing vessels engaged in fishing per day from 2015 through 2017. The grey shading shows the yearly Chinese moratoria, from May 16^*th*^ to September 16^*th*^ in 2015 and 2016, from May 1^*st*^ to September 16^*th*^ in 2017. (B-I) relative variation of fishing effort $E_{g}/{\bar{E}}_{g}$, not including the Chinese EEZ.

Over the observed period, trawling accounts for about half (53%) of the total AIS effort, while fixed gears, drifting gears, purse seiners and squid jiggers account for 15%, 13%, 11 and 4% respectively. The global time-series of *E* = ∑_*g*_ *E*_*g*_ shows marked seasonal variation with a large decrease in fishing effort during the boreal summer (see Fig 1A). The dominant yearly signal has been previously attributed to variations of effort in the Chinese Exclusive Economic Zone (EEZ) driven by annual fishing moratoria during northern hemisphere summer months [25]. When we remove the Chinese EEZ, the resulting global time-series of ∑_*g*_ *E*_*g*_ indicates a relatively constant effort over time (see S2 Fig). Reductions in effort occur repeatedly during December, January and February (Fig 1A), which were attributed by ref. [25] to the Christmas, Occidental New Year and Chinese New Year holidays, respectively. We estimate the implied number of holidays by computing the deviation from the average total monthly effort from March through November (not including the Chinese EEZ), arriving at 4.0 days in December, 4.0 in January and 2.5 in February. We have no way to verify that holidays are the only reason for the reduced effort during these boreal winter months, but the number of implied holidays is not inconsistent with typical holiday breaks in the industrialized world.

Looking by gear type $E_{g}/{\bar{E}}_{g}$ (Fig 1B–1I) we see that, when data from the Chinese EEZ is removed, the pronounced minimum during boreal winter is the main source of variation of summed effort (Fig 1B). The boreal winter minimum is particularly dominant for trawlers, fixed gears and coastal purse seiners (Fig 1C, 1E and 1G). Moreover, the monthly time-series show that trawls, drifting longlines, fixed gears and high seas purse seines are deployed at a relatively constant rate throughout the global ocean over the year (Fig 1C–1F), with slight oscillations of ±15% around the mean (standard deviations are, in increasing order, $sd(E_{Drifting}/{\bar{E}}_{Drifting})≃6\%$, $sd(E_{Trawled}/{\bar{E}}_{Trawled})≃9\%$, $sd(E_{Purse\;(high\;seas)}/{\bar{E}}_{Purse\;(high\;seas)})≃13\%$ and $sd(E_{Fixed}/{\bar{E}}_{Fixed})≃15\%$). Because these gear types dominate the GFW effort, the total effort is relatively constant. In contrast, the effort of coastal purse seiners and squid jiggers show larger amplitudes in monthly integrated effort (Fig 1G and 1I), reaching up to ±50% of the mean ($sd(E_{Purse\;(coast)}/{\bar{E}}_{Purse\;(coast)})=0.25$ and $sd(E_{Jigger}/{\bar{E}}_{Jigger})=0.25$).

Summed globally, the fishing effort varies strongly, but most of this variability comes from societal drivers in the form of regulation (the Chinese moratorium) and holidays. From this point further, we analyze fishing effort once the Chinese EEZ is removed.

### Spatial patterns of effort seasonality

**Fig 2:**
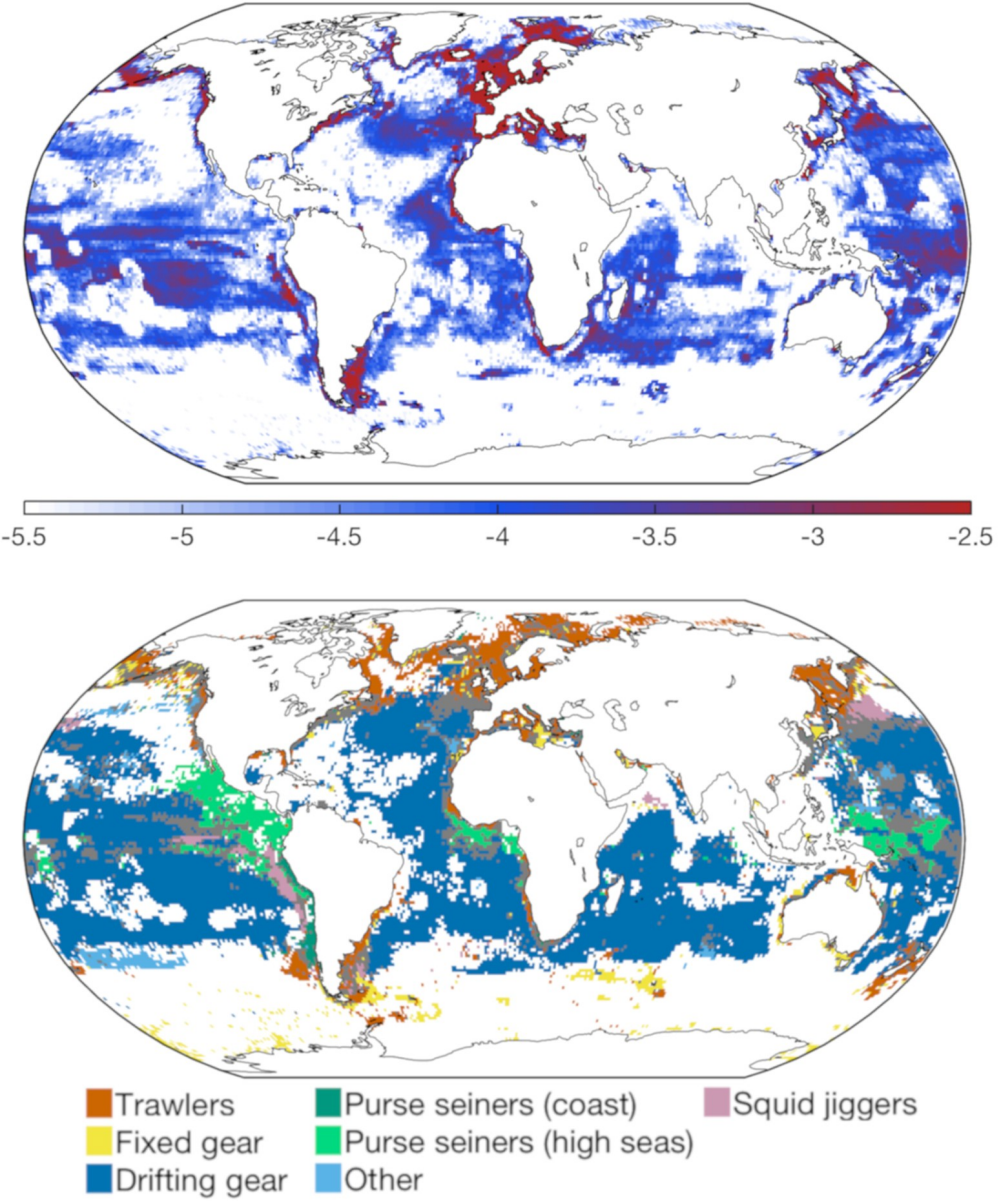
Spatial distribution of observed fishing effort. (A) mean local monthly fishing effort ${\bar{e}}^{i,j}$ over the years 2015 through 2017, on a *log*10 scale (in *Fishing days*/*month*/*km*^2^), on a 1.0° grid; (B) main gear type used in different regions. A gear type is considered dominant when a single fishery represents more than 75% of the summed fishing effort in a 1.0° cell. Grey areas correspond to cells where no gear type is clearly dominant.

Next, we look at the spatial distribution of effort and its seasonal variation. We refer to the local fishing effort at each geographical location (*i*, *j*) as $e_{g}^{i,j}$ for a gear type *g*. The local total fishing effort $e^{i,j}=\sum {}_{g}e_{g}^{i,j}$ is very heterogeneous, with the average monthly effort, ${\bar{e}}^{i,j}$, spanning more than 3 orders of magnitude between intensely-fished coastal regions and low-effort expanses of the high seas (Fig 2A). The distribution of gear types is broadly related to the intensity of effort (compare Fig 2A with Fig 2B), with intensely-fished regions (mostly on continental shelves) being dominated by trawled and fixed gears, and the least frequently fished regions (mostly on the high seas) being dominated by drifting gears and purse seiners.

**Fig 3:**
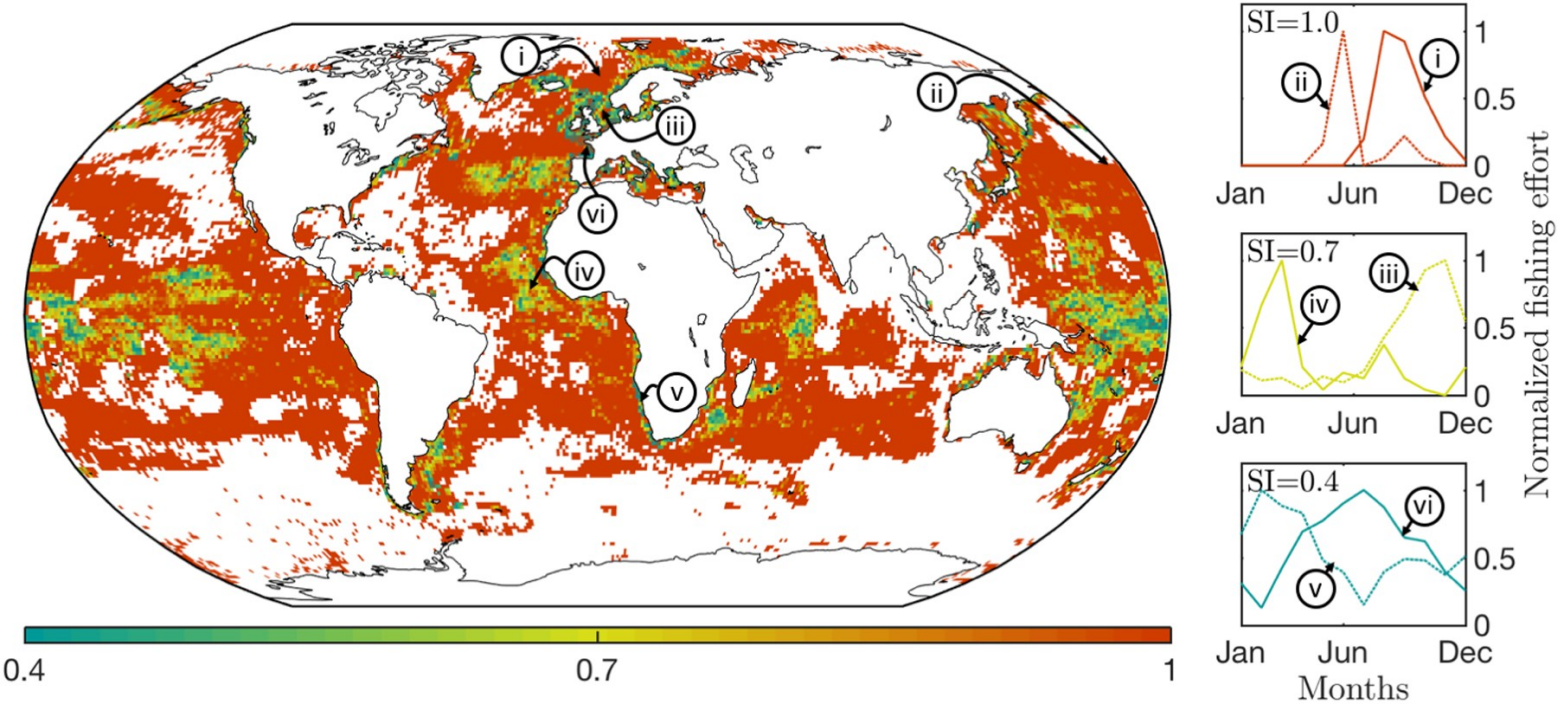
Spatial distribution of the seasonality indicator. The seasonality indicator *SI*^*i*,*j*^ reflects how effort is distributed during the year: *SI*^*i*,*j*^ < 0.4 only slight variations; *SI*^*i*,*j*^ ≃ 0.7, markedly higher effort during some portion of the year; *SI*^*i*,*j*^ > 1.0, most effort invested within a consistent 2–3 months interval. The same value of *SI*^*i*,*j*^ can correspond to different patterns, see figures on the right, from top to bottom *SI*^*i*,*j*^ ≃ 1.0, 0.7 and 0.4. The indicator *SI*^*i*,*j*^ is computed on a 1.0° grid.

In order to explore how the intensity of seasonal cycles of local fishing effort varies, we use the seasonality indicator *SI*^*i*,*j*^, and calculate it at all (*i*, *j*) locations over the three years of GFW data (Materials and Methods). A low value of *SI*^*i*,*j*^ (*e.g*. < 0.4) indicates that exploitation occurs consistently throughout the year. In contrast, a high value of *SI*^*i*,*j*^ (*e.g*. > 1.0) indicates that fishing activity occurs during only a few months of the year, with little or no fishing activity registered during the rest of the year. As shown in Fig 3, large regions of the open ocean are only exploited during 2 to 3 months every year (*SI*^*i*,*j*^ > 1.0) while the highest concentrations of effort in high-latitude coastal seas are generally associated with a more constant level of exploitation (*SI*^*i*,*j*^ < 0.4). Qualitatively, the pattern of *SI*^*i*,*j*^ is similar to that of the standard deviation (see S3 Fig). Since the analysis is based on the presence/absence of effort to estimate seasonality, it is sensitive to grid resolution, which alters the distribution of *SI*^*i*,*j*^ slightly (see S4 Fig). However, it appears that most of the vast expanse of the open ocean is visited only during certain times of the year.

**Fig 4:**
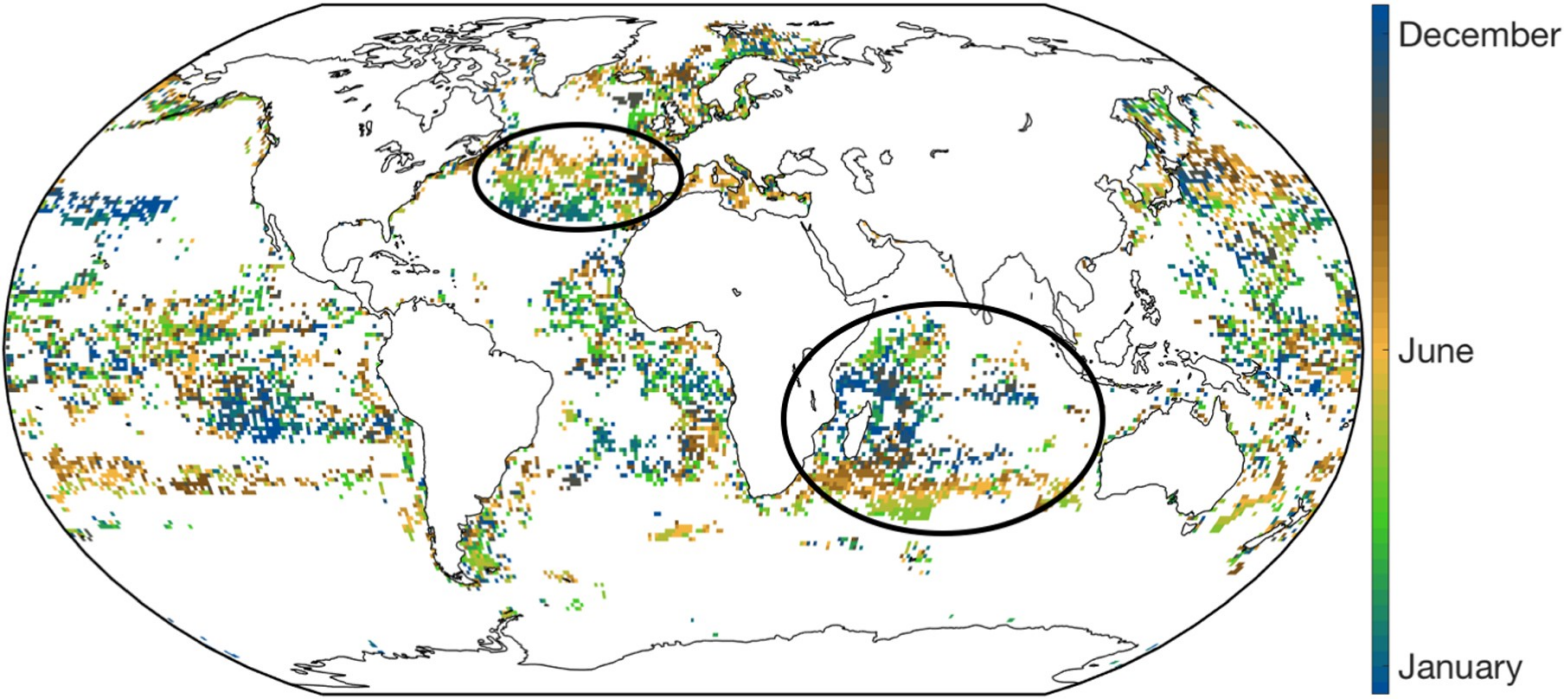
Month during which a seasonal local maximum of fishing effort occurs. The month of the peak of fishing effort *M*_*max*_ is determined from the climatological first principal component in all grid cells for which a clear seasonal cycle is identified. Regions with coherent patterns explained in the discussion are circled. Analysis conducted on a 1.0° grid.

Fig 4 shows the month of the year during which seasonal effort is highest. In order to limit this to cells in which there is a relatively clear and identifiable seasonal cycle, we only show grid cells for which (1) the total effort $e^{\left(i,j\right)}=\sum_{g}e_{g}^{\left(i,j\right)}$ from 2015 to 2017 is nonzero for all three years, leaving 97% of cells on a 1.0° grid, and (2) the first principal component time-series explained more than 2/3 of the local effort variations, leaving 76% of cells. Because Fig 4 is sensitive to grid resolution, we show the same quantity calculated at other resolutions in supplementary S4 Fig. Some parts of the ocean show little coherence, with neighboring grid cells peaking in very different months (*e.g*. most of the West tropical Pacific). We interpret this lack of coherence as indicating regions with either multiple seasonal peaks that our method cannot differentiate, or spurious seasonal peaks that are statistical artifacts. In contrast, some open-ocean regions show coherent patterns of fishing effort, particularly in regions with a pronounced seasonal distribution of effort (*i.e*. a high *SI*^*i*,*j*^ Fig 3, compare with *M*_*peak*_ homogeneity Fig 4). Coherent patterns are particularly visible in the Indian Ocean, where effort shifts south or north of the central basin during austral summer, and in the North Atlantic, where effort progressively shifts from lower to higher latitudes from boreal spring to summer.

### Seasonality and distance to port

**Fig 5:**
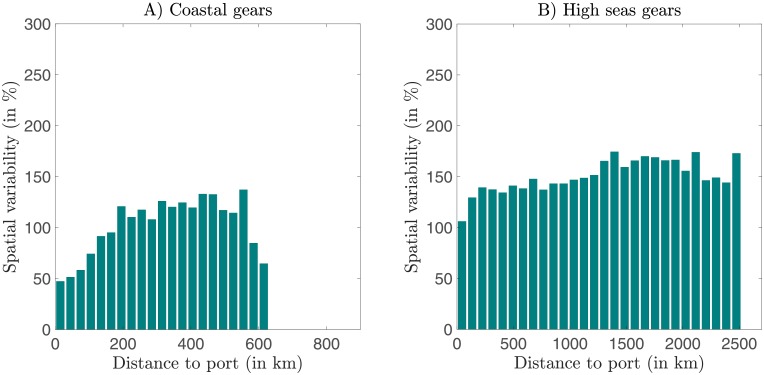
Temporal variability of effort at increasing distance from the nearest port. (A) Gears targeting fish in coastal ecosystems: trawlers, fixed gears and coastal purse seiners. (B) Gears deployed anywhere: drifting longlines, high seas purse seiners and squid jiggers. The variability is computed as the mean local standard deviation $\bar{sd(e_{g}^{i,j}/{\bar{e}}_{g}^{i,j})}$ per distance bins weighted by the mean local monthly effort over the years 2015 through 2017. Distance bins with a very low fishing effort have been removed (less than 0.1% of global effort).

Next, we characterize how the seasonality of fishing effort varies as a function of the distance to nearest ports. The local relative standard deviation of monthly effort, $sd(e_{g}^{i,j}/{\bar{e}}_{g}^{i,j})$, averaged per distance bin *b* once weighted with ${\bar{e}}_{g}^{i,j}$, increases with distance to port, for all gear type (S5 Fig). However, the rate of increase differs between gears. For coastal gears, namely trawlers, fixed gears and coastal purse seiners, which fish at less than 600 km from nearest port, the increase of variability is almost 3-fold (Fig 5A). This suggests that the most temporally-constant fishing activity by coastal gears occurs in fishing grounds that can be conveniently accessed from ports. In contrast, the high seas gears, drifting longlines, high seas purse seiners and squid jiggers, do not show a limit to their distance from port, but occur at every possible distance from port. Despite the much greater range of distances accessed by high seas gears, the increase of temporal variability from nearest to furthest fishing grounds is less pronounced and occurs mostly within the first 100 km (Fig 5B). Thus, the temporal variability of effort by high seas gears appears to be much less strongly related to the accessibility of fishing grounds to port.

### Overview of seasonality by gear type

Finally, we summarize how the seasonality of effort at the local scale compares to the seasonality of the globally-integrated effort for each gear type. We calculate the local relative standard deviation of monthly effort, $sd(e_{g}^{i,j}/{\bar{e}}_{g}^{i,j})$, where ${\bar{e}}_{g}^{i,j}$ is the local annually-averaged effort of gear type *g*. We then take the global mean, weighted with ${\bar{e}}_{g}^{i,j}$, to provide an indication of the spatial variability that is characteristic of fishing gear *g* the world over. We compare this with the seasonal variability of integrated global effort (Fig 1), characterized as $sd(E_{g}/{\bar{E}}_{g})$.

**Fig 6:**
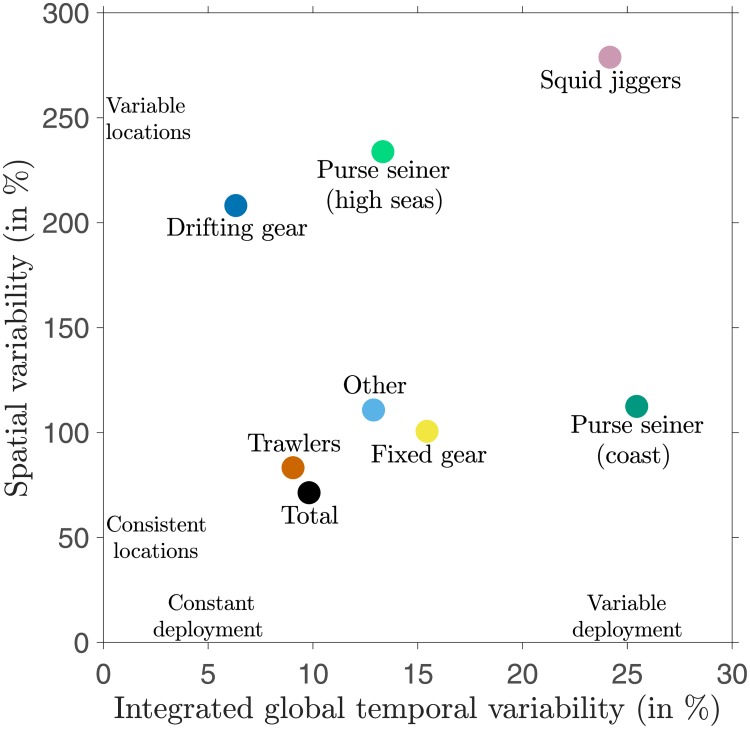
Seasonality in spatial distribution vs. global deployment of effort. The seasonality in spatial distribution is computed as the mean local standard deviation $\bar{sd(e_{g}^{i,j}/{\bar{e}}_{g}^{i,j})}$ when weighted by the mean local monthly effort over the years 2015 through 2017. The seasonality in global deployment is computed as the global standard deviation $sd(E_{g}/{\bar{E}}_{g})$ over the years 2015 through 2017.

The resulting patterns (Fig 6) show clear distinctions in the seasonality of effort among fisheries. Note that these clear distinctions are conserved when the seasonality of effort at the local scale is computed on different grid resolutions (see S4 Fig). The global effort associated with drifting longlines is the most constant ($sd(E_{Drifting}/{\bar{E}}_{Drifting})≃6\%$) but the spatial variability is high ($\bar{sd(e_{Drifting}^{i,j}/{\bar{e}}_{Drifting}^{i,j})}≃210\%$). Thus, drifting longlines are deployed throughout the year in the global ocean, but their spatial distribution shifts strongly over time. Trawled gears are also deployed with a similar constancy of integrated global effort, but there is less spatial variability ($\bar{sd(e_{Trawled}^{i,j}/{\bar{e}}_{Trawled}^{i,j})}<100\%$). Fixed gears are deployed with a spatial consistency that is similar to trawled gears, but with more integrated temporal variability. Purse seiners show distinct characteristics in high seas versus coastal regions, with more consistent spatial deployment in coastal regions ($\bar{sd(e_{Purse\;(coastal)}^{i,j}/{\bar{e}}_{Purse\;(coastal)}^{i,j})}≃100\%$ vs. $\bar{sd(e_{Purse\;(high\;seas)}^{i,j}/{\bar{e}}_{Purse\;(high\;seas)}^{i,j})}≃200\%$), but more constant globally-integrated effort in the high seas ($sd(E_{Purse\;(high\;seas)}/{\bar{E}}_{Purse\;(high\;seas)})≃13\%$ vs $sd(E_{Purse\;(coastal)}/{\bar{E}}_{Purse\;(coastal)})≃25\%$). For squid jiggers the effort is highly variable both seasonally and spatially.

## Discussion

Our results show that the total global industrial fishing effort, as revealed by AIS, follows a strong seasonal cycle (Fig 1). The most important contributor to this seasonality is the annual Chinese fishing moratorium. If the Chinese EEZ is removed, the integrated global effort is relatively constant throughout the year for most gear types, apart from a minimum from December to February that can be largely attributed to seasonal holidays. The main exceptions to this constancy are coastal purse seiners and squid jiggers, for which the integrated effort changes by a factor of two between seasons. More important differences between gear types are found when we consider spatial variability in seasonal effort (Fig 6). Coastal gears tend to fish consistently in the same waters throughout the year, especially close to port. In contrast, gears deployed on the high seas show pronounced seasonal movements between regions, and are less dependent on the distance to port.

Given that the industrial fishery is almost entirely profit-driven, we presume that these gear-specific distributions of fishing effort reflect variability (or the absence thereof) in profit over the seasonal cycle. For example, the weak seasonality of trawlers operating close to port suggests that the expected revenue remains consistently larger than marginal costs throughout the year, so that it is always economically-rational to fish at a similar level. This small variability could be due to the dominance of fixed costs (*e.g*. financing and depreciation of vessels) and/or subsidies of marginal costs (*e.g*. fuel), but the implied weak seasonality in revenue also suggests an availability of prey throughout the year.

Below, we consider the potential drivers of variability in the bioeconomic system in the context of prior work on local fisheries. Because different gears are deployed by different vessel types and target different ecosystems (Fig 2B), they are likely to have different sensitivities to natural seasonal variations and to be subject to different socio-economic influences. We subsequently discuss these expected gear-specific features.

### Drivers of fishing seasonality

To aid in discussing the diverse possible drivers behind seasonal variations among the thousands of fisheries included in this global perspective, we assume that effort approximately evolves as a function of profit and regulation:

$$\frac{dE}{dt}\propto \kappa_{e}\left(p\frac{H}{E}-c\right)-R$$ (2)

where *E* is effort (in the GFW data in *Fishing days*/*month*/*km*^2^), *p* is the ex-vessel price (in $/*kg*) of *H* fish harvested (in *kg*/*month*/*km*^2^), *c* is the cost per unit effort (in $/*Fishing day*), and *R* is the reduction of effort by regulation (in *Fishing days*/*month*/*km*^2^). The harvest is assumed to vary as *H* = *qEB*, where *q* is an inherent catchability for a gear / fish combination (in *km*^2^/*Fishing day*), and *B* is the local biomass density (*kg*/*km*^2^). This simple equation ignores complex aspects of fleet composition and adjustment, and assumes rational profit-seeking behavior, but nonetheless provides a conceptual framework within which to consider processes of first-order importance. We consider, in turn, how each variable, socio-economic or natural, might contribute to seasonality.

The price, *p*, could vary seasonally due to changes in seasonal production and demand [18, 30]. At stock level, seasonal cycles in fish condition may also influence the quality of fish and its price [31, 32].

The cost, *c*, might vary seasonally, such as in response to the difficulty of operating during difficult weather conditions [33]. In our formulation, the cost also includes the cost of travel from port to the fishing location, thus if prey move seasonally to a distance from port that is more costly to access it could decrease effort [10].

Regulations, *R*, are certainly an important driver as most notably illustrated by the dominant effect of the above-mentioned Chinese moratoria. Seasonal regulations include spatial closures at stock level, within EEZ or in the high seas [34–36], affecting selected gears or all of them. They also include seasonal fishing quotas [18].

Seasonal changes in *B* could arise from local changes in stock-level biomass production rates (somatic growth), and can also be affected by the movement of fish. The degree to which biomass production might alter *B*, such as due to seasonality of primary production, can be gauged by considering the typical size/weight/age at which fish are caught. Numerical experiments using size-spectrum models show that seasonal somatic growth and reproductive cycles generate waves of stock biomass propagating from smaller to larger individuals’ sizes and dampening after a maximum at a “resonant size/weight”. In these experiments, the maxima of seasonal biomass *B* variations occur for individuals less than one year old, which are less than 20 cm long [37] and weight less than 10 g [38]. The integration of growth over multiple years causes larger individuals to buffer seasonal variations of *B*. This sensitivity of biomass to annual cycles will be modulated by the life history of different species. For example, cephalopods, with very fast life cycles, respond particularly strongly to seasonal variations of production [39]. The second potential source of variability in *B*, fish movement, can occur through seasonal migrations for reproduction [40] or seasonal contraction of habitat [2, 17], both of which can drive a change from widely dispersed to concentrated. Thus, fish movement can drive local seasonal increases of *B* for the same overall population size.

Seasonal changes in *q* can arise from seasonal changes in fish behavior that alter their seasonal availability to a specific fishing gear. Aggregation drivers can lead to schooling [41], facilitating capture with net, or a greater propensity to bite hooks, which can both increase *q*.

### Gear-specific sensitivity to seasonality drivers

Trawlers and fixed gears both showed very little seasonality in effort (Fig 6), target similar regions (Fig 2B), and what seasonality they do show is strongly linked to distance to port (Fig 5A). We suggest that a first reason for the weak variability is that both of these coastal gear types can apply generalist strategies, targeting multiple species *s* [11, 18, 42]. Each of these prey items follow seasonal cycles such as migrations or aggregations, thus, for each species *q*_*s*_ and *B*_*s*_ is expected to vary seasonally to some degree [9, 10]. However, in a given region, the variation in *q*_*s*_ and *B*_*s*_ can be asynchronous due to species-dependent variations in life history strategies and behavior. Generalist strategies take advantage of these asynchronous variations such that the total catchability *q* = ∑ *q*_*s*_ and biomass available *B* = ∑ *B*_*s*_ are smoothed, ensuring a relatively continual availability of prey [43]. A second reason is the more sedentary nature of coastal communities attached to the continental shelf. At a coarse resolution of 1°, the local variation in *q*_*s*_ and *B*_*s*_ due to migration appears smoothed for these species since they swim on smaller distances. This would also contribute to a relative continual availability of prey to fishers. The fact that variability increases with increasing distance to port is consistent with the increased costs of traveling to more distant locations, resulting in a requirement for higher marginal revenues, so that fishing effort in more distant waters has a greater sensitivity to seasonal changes in *q* and *B*. Finally, while we are unaware of seasonal price *p*_*s*_ variations, seasonal stocks regulations *R*_*s*_ influence many high value species [18, 34]. Seasonal regulations *R* = ∑ *R*_*s*_ or cost *c* variations of different value species must then be incorporated into fishers’ strategy to ensure a more continuous revenue [18, 44], leading locally to a constant fishing effort.

In contrast with the coastal gears, the high seas drifting longlines and purse seiners both show significantly more seasonality in locations fished, but with a weak dependence on distance from port (Fig 5B). Cost is likely to contribute to the relatively weak dependence on distance, since these gears target high value pelagic species such as tuna or billfishes [40, 45] and incur high fixed costs relative to operation costs [46]. Because fixed costs such as capital investments and gear maintenance have no seasonality, their dominance would be expected to dampen the range of total cost variations associated with operation, and the main consideration for operators would be to ensure sufficient revenue throughout the year to cover the fixed costs. The seasonality in spatial deployment of these gears is unlikely to be related to price, since the target species are generally traded in a global market and prices don’t follow a seasonal pattern in ref. [30]. Meanwhile, regulation with seasonal closures are implemented in the high seas, but to our knowledge they cover small regions compared to the habitat range of pelagic species and are applied for a short time [35, 36], including the difficulty of regulating high seas fisheries [47] it seems unlikely that they drive the observed seasonality of these gears on the high seas. In contrast to the absence of obvious seasonal socio-economic drivers, seasonal changes in *B* and *q* are likely to be important drivers of seasonality in the high seas effort (Fig 6). Since longliners specialize in larger sizes (average length of 115 cm according to ref. [48]), seasonal variations of biomass production *B* are unlikely to have an influence, given the size-dependent dampening of seasonality discussed above. Therefore, fish behavior is likely to dominate the strong spatial variability of longline fishing effort, consistent with the patterns observed Fig 4 (see the regions circled). For example, in the North Atlantic, the northern shift of the fishing effort of drifting longlines during boreal summer corresponds to the northern shift of Bluefin tuna stocks [49], while in the Indian Ocean, the concentration of fishing effort in the central basin from November to January, as well as the seasonal shifts towards the north and south the rest of the year, correspond to the migrations of albacore tuna [40]. The fact that high seas purse seines often target smaller individuals [48], allows for the possibility that biomass production cycles also contribute to the seasonality, in addition to migration and behavioural changes.

Coastal purse seines provide a special case of coastal gears. Coastal purse seines generally depend on the seasonal migration of schooling pelagic prey into coastal fishing grounds, and are therefore sensitive to the local variation of biomass *B* and catchability *q* of target species. Because purse seine fisheries often target only one or two species of small individuals with strong seasonality (e.g. Peruvian anchoveta [6]) they are more likely to be deployed during only part of the year. The relative consistency in deployment location (Fig 6) can be explained by predictable seasonal migrations of schooling fish to regular fishing grounds.

Similarly, squid jiggers present a special case of high seas gears. Unlike the tuna and billfish generally targeted by drifting longlines and high seas purse seines, squid are fast-living species with an annual life cycle [8, 39]. Large seasonal variations of biomass production *B* are therefore expected. We suggest that the strong seasonality of squid jigger effort (Fig 6), with global peaks during March-May and September-November (Fig 1I), is consistent with the peaks of biomass production in the main fishing grounds of the Northwest Pacific, the Humboldt current and the Patagonian shelf (Fig 2B).

### Limits of data coverage

Given the nature of the GFW data, our analysis is limited to industrial fishing, and is biased toward the high seas and wealthier nations. Thus, we may underestimate the true difference of intensity between spatially variable and spatially consistent gears. Moreover, small-scale coastal fisheries, which constitute a large fraction of global fish catches and a large proportion of all fishers [50], may follow very different patterns of seasonality from those described here. Although we do not have wide-scale observations for small scale fisheries that are comparable to the GFW data, prior work has shown that they commonly show greater diversification than the large industrial vessels discussed here. This diversification helps to buffer against seasonal variations of prey availability and weather conditions, and to maintain activity throughout the year [13, 33, 43]. Thus, although seasonal prey-switching is likely very important, we hypothesize that small-scale fisheries will show weak seasonality in both their total effort and its spatial distribution.

## Conclusion

Here we have provided the first detailed global analysis of seasonal variability in fishing effort, using the GFW data from 2015 through 2017. Apart from the strong impact of the yearly closure of the Chinese fishery and global cultural habits such as holidays, the total industrial fishing effort shows modest seasonality. Our results reveal that trawlers, the dominant gear type in the dataset, regularly visit the same regions with high intensity. These vessels, although targeting seasonally moving species, show consistent total levels of effort and no clear sign of seasonality. Fixed gears have similar characteristics. In contrast, the high seas purse seiners and drifting longliners both show high spatial variability in their distribution of effort. Like other large pelagic predators, they move seasonally in search of high densities of catchable biomass. For these gears, we suggest that a main factor driving this seasonal range shift is fish behavior: fishing effort is focused where fish of sufficient size aggregate seasonally in high densities and/or when their behavior makes them easier to catch, dependent on gear type. Unlike the other gears, coastal purse seiners and squid jiggers display significant seasonal variations of the total deployed effort. We suggest seasonal migrations of prey into fishing grounds, and seasonal variations of biomass production are the main driver for these specialized gears. Thus, while the total fishing effort is dominated by seasonal socio-economic drivers, spatial seasonal variations can be explained by natural seasonal drivers.

It is still early days for GFW, with detailed data only becoming widely publicly available in 2018. As the database grows, and coverage of vessels becomes more complete, it will be possible to observe many more details than can be resolved at present. The study of global fishing effort is therefore on its way to become a remarkably data-rich example of human-ecosystem interaction.

## Supporting information

S1 Fig Distance to nearest port. The distance to the nearest port in *km* of the center of ocean cells on a 1° grid as interpolated from a 1/100° map developed by Global Fishing Watch.
(PDF)

S2 Fig Global mean monthly effort *E* without Chinese EEZ. Monthly mean number of fishing days normalized by the number of days for each month, reflecting the mean number of fishing vessels engaged in fishing per day from 2015 through 2017. This effort is after removal of the Chinese EEZ.
(PDF)

S3 FigAlternative measure of the spatial distribution seasonality.Spatial distribution of the standard deviation of local effort relative variation. The standard deviation of the relative variation of effort $sd(e^{i,j}/{\bar{e}}^{i,j})$ is computed in each ocean cell from 2015 through 2017, in % of the mean.(PDF)

S4 FigEffect of grid resolution.(a) Spatial distribution of seasonality indicator *SI* at different grid resolutions (top 0.5° and bottom 4.0°). (b) Months of the peak of fishing effort in all grid cells where a seasonal cycle is clearly identified for different grid resolutions (top 0.5° and bottom 4.0°). (c) Mean local standard deviation $\bar{sd(e_{g}^{i,j}/{\bar{e}}_{g}^{i,j})}$ computed when effort is binned on different grid resolutions.(PDF)

S5 FigVariability and distance to port per gear type.Mean local standard deviation $\bar{sd(e_{g}^{i,j}/{\bar{e}}_{g}^{i,j})}$ per 75 km distance bin to the nearest port, over the years 2015 through 2017, per fishing gear, in %.(PDF)
