# Supplementary material for: Seasonal variability in global industrial fishing effort
Source: PLoS One. 2019 May 17;14(5):e0216819. doi: 10.1371/journal.pone.0216819 (PMC6524810; doi:10.1371/journal.pone.0216819)
Supplement: S1 Fig — The distance to the nearest port in km of the center of ocean cells on a 1° grid as interpolated from a 1/100° map developed by Global Fishing Watch. (PDF) [file pone.0216819.s001.pdf]

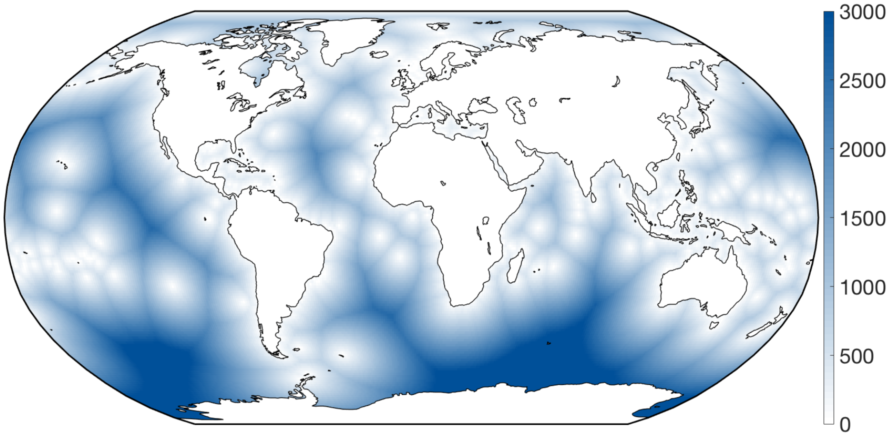

S1 Fig. Distance to nearest port. The distance to the nearest port in km of the center of ocean cells on a 1° grid as interpolated from a $1/100^o$ map developed by Global Fishing Watch.
