# Supplementary material for: Seasonal variability in global industrial fishing effort
Source: PLoS One. 2019 May 17;14(5):e0216819. doi: 10.1371/journal.pone.0216819 (PMC6524810; doi:10.1371/journal.pone.0216819)
Supplement: S2 Fig — Monthly mean number of fishing days normalized by the number of days for each month, reflecting the mean number of fishing vessels engaged in fishing per day from 2015 through 2017. This effort is after removal of the Chinese EEZ. (PDF) [file pone.0216819.s002.pdf]

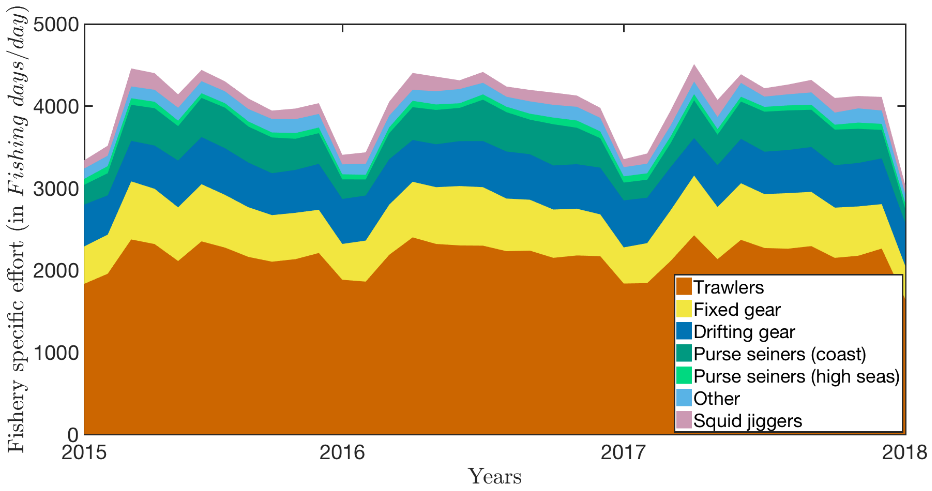

S2 Fig. Global mean monthly effort E without Chinese EEZ. Monthly mean number of fishing days normalized by the number of days for each month, reflecting the mean number of fishing vessels engaged in fishing per day from 2015 through 2017. This effort is after removal of the Chinese EEZ.
