# Supplementary material for: Seasonal variability in global industrial fishing effort
Source: PLoS One. 2019 May 17;14(5):e0216819. doi: 10.1371/journal.pone.0216819 (PMC6524810; doi:10.1371/journal.pone.0216819)
Supplement: S3 Fig — Spatial distribution of the standard deviation of local effort relative variation. The standard deviation of the relative variation of effort sd(ei,j/e¯i,j) is computed in each ocean cell from 2015 through 2017, in % of the mean. (PDF) [file pone.0216819.s003.pdf]

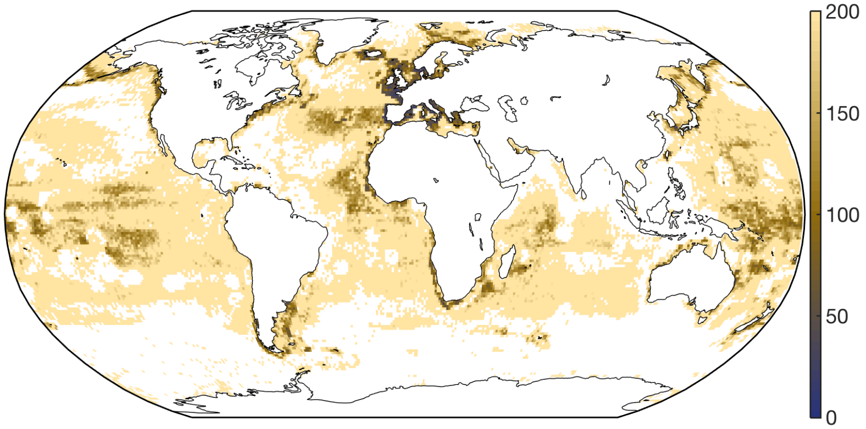

S3 Fig. Alternative measure of the spatial distribution seasonality. Spatial distribution of the standard deviation of local effort relative variation. The standard deviation of the relative variation of effort $sd(e^{i,j}/\overline{e}^{i,j})$ is computed in each ocean cell from 2015 through 2017, in % of the mean.
