# Supplementary material for: Seasonal variability in global industrial fishing effort
Source: PLoS One. 2019 May 17;14(5):e0216819. doi: 10.1371/journal.pone.0216819 (PMC6524810; doi:10.1371/journal.pone.0216819)
Supplement: S4 Fig — (a) Spatial distribution of seasonality indicator SI at different grid resolutions (top 0.5° and bottom 4.0°). (b) Months of the peak of fishing effort in all grid cells where a seasonal cycle is clearly identified for different grid resolutions (top 0.5° and bottom 4.0°). (c) Mean local standard deviation sd(egi,j/e¯gi,j)¯ computed when effort is binned on different grid resolutions. (PDF) [file pone.0216819.s004.pdf]

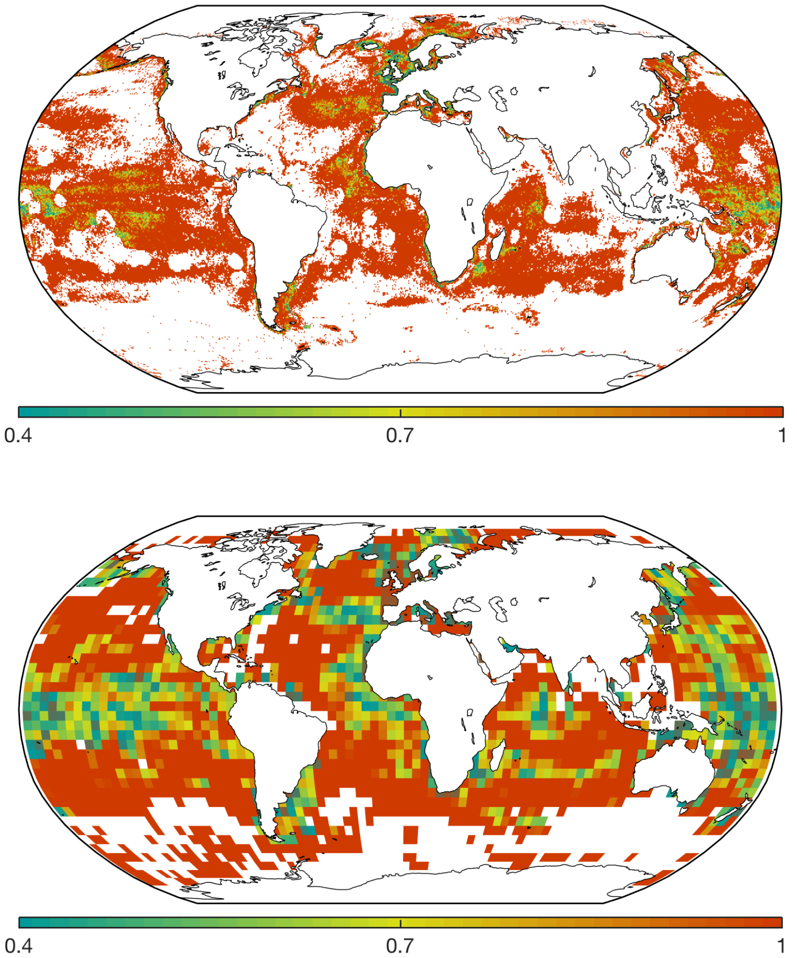

S4 Fig. Effect of grid resolution. (a) Spatial distribution of seasonality indicator SI at different grid resolutions (top 0.5^o and bottom 4.0^o).

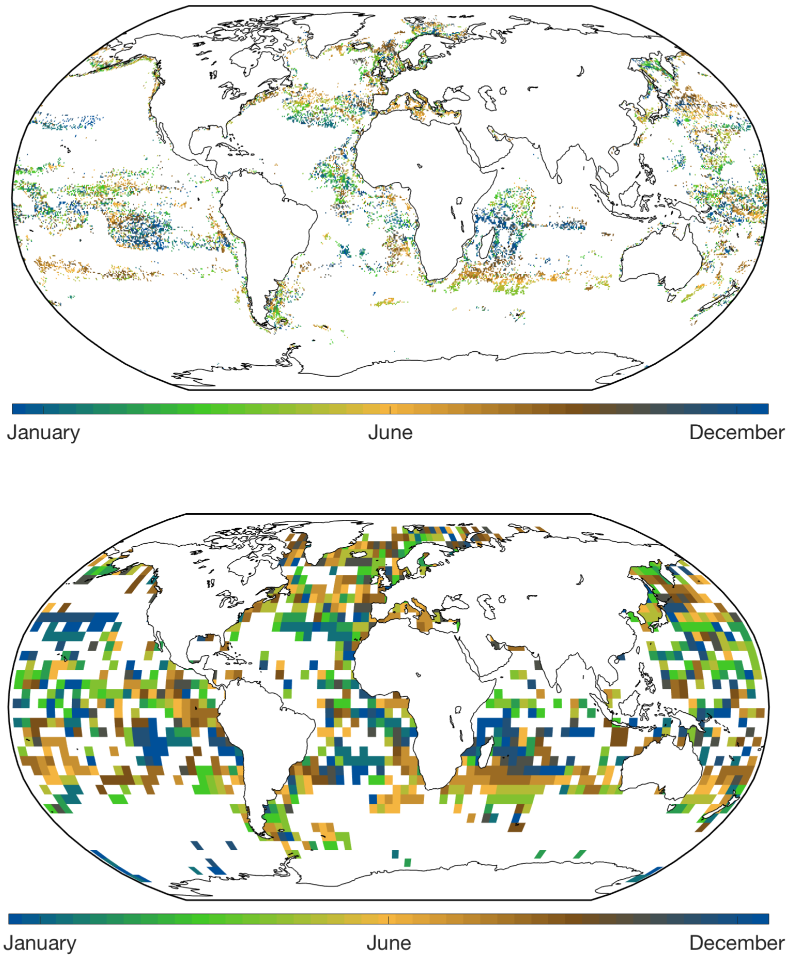

S4 Fig. Effect of grid resolution. (b) Months of the peak of fishing effort in all grid cells where a seasonal cycle is clearly identified for different grid resolutions (top 0.5^o and bottom 4.0^o).

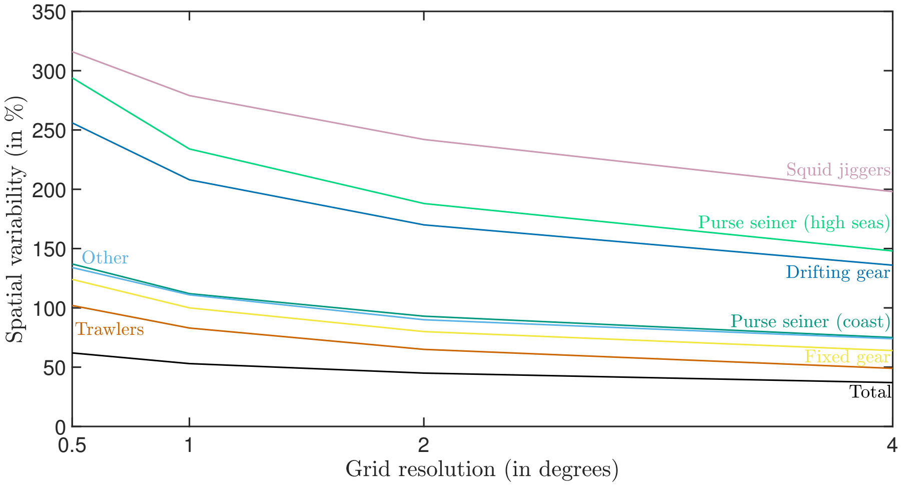

S4 Fig. Effect of grid resolution. (c) Mean local standard deviation $\overline{sd(e_g^{i,j}/\overline{e}_g^{i,j})}$ computed when effort is binned on different grid resolutions.
