# Supplementary material for: Seasonal variability in global industrial fishing effort
Source: PLoS One. 2019 May 17;14(5):e0216819. doi: 10.1371/journal.pone.0216819 (PMC6524810; doi:10.1371/journal.pone.0216819)
Supplement: S5 Fig — Mean local standard deviation sd(egi,j/e¯gi,j)¯ per 75 km distance bin to the nearest port, over the years 2015 through 2017, per fishing gear, in %. (PDF) [file pone.0216819.s005.pdf]

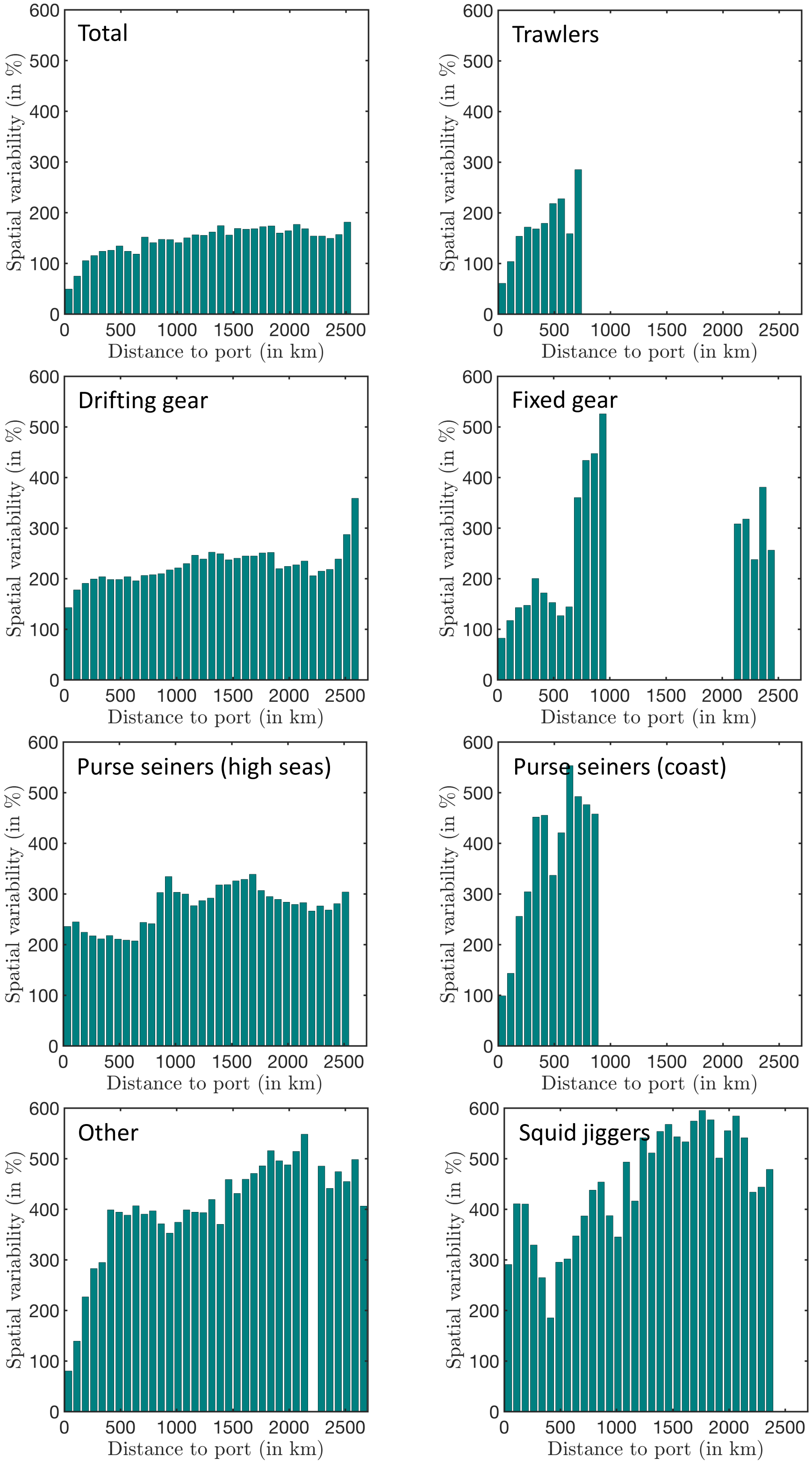

S5 Fig. Variability and distance to port per gear type. Mean local standard deviation $\overline{sd(e_g^{i,j}/\bar{e}_g^{i,j})}$ per 75 km distance bin to the nearest port, over the years 2015 through 2017, per fishing gear, in %.
